# Analysis of balance in adults with chronic axial spinal pain receiving medial branch block or radiofrequency ablation procedures

**DOI:** 10.1002/pmrj.70083

**Published:** 2026-01-19

**Authors:** Matthew Carter, John D Ralston, Robert Burnham, Jennifer Laxshimalla, Lisa Jasper, JuliAnn Thai, Ashley D Smith

**Affiliations:** ^1^ Department of Medicine Faculty of Medicine & Dentistry, University of Alberta Edmonton Alberta Canada; ^2^ Neursantys Inc Calgary Alberta Canada; ^3^ VivoCura Health Calgary Alberta Canada; ^4^ Central Alberta Pain and Rehabilitation Institute Lacombe Alberta Canada; ^5^ Department of Clinical Neurosciences Cumming School of Medicine, University of Calgary Calgary Alberta Canada; ^6^ Department of Physical Therapy Faculty of Rehabilitation Medicine, University of Alberta, Edmonton, Alberta, Canada Edmonton Alberta Canada

## Abstract

**Background:**

Medial branch blocks (MBB) and radiofrequency ablations (RFA) are common diagnostic, prognostic, and interventional procedures performed for the management of cervical and lumbar spine facetogenic pain. Although these procedures have established utility as pain management strategies, it is unclear if the disruption in function of the medial branch nerve leads to patient balance impairment.

**Objective:**

(1) To determine if there was a difference in postural balance, measured using a head mountable physiological vibration acceleration (Phybrata) sensor, between those pursuing cervical or lumbar MBBs/RFAs compared to an asymptomatic healthy cohort (HC). (2) To determine differences in balance and sensory reweighting prior to and following MBBs/RFAs, and the association with clinical measures of pain and balance using the 4‐Stage Balance Test.

**Design:**

Quasiexperimental study with matched controls.

**Setting:**

Community multidisciplinary chronic musculoskeletal pain practice.

**Participants:**

Sixty individuals with chronic axial back pain and 60 age‐/gender‐matched healthy individuals.

**Interventions/main outcome measures:**

Standing balance measures recorded with the Phybrata sensor, subjective report of pain (numerical rating scale), and clinical measures of balance (4‐Stage Balance Test) were analyzed before and after participants underwent MBBs/RFAs and compared to an asymptomatic HC.

**Results:**

Participants with axial spinal pain of facet joint origin (≥ 50% relief post MBB/RFA) demonstrated balance degradation when standing with eyes closed, when compared to participants with nonspecific axial spinal pain (< 50% pain relief) or HCs – both prior to and following MBBs/RFAs. Following MBBs/RFAs, participants undergoing C2/3 procedures demonstrated increased postural sway. The remainder of participants with axial spinal pain did not show any further balance deficits, with corresponding sensory reweighting adaptation. Phybrata measures were not associated with pain location or pain intensity prior to or after MBBs/RFAs.

**Conclusions:**

These results suggest the risk of developing significant balance impairments following MBB/RFA procedure of either the cervical (excluding C2/3) or lumbar spine is low, although residual balance deficits remain.

## INTRODUCTION

Axial spinal pain is a common cause of morbidity and contributes to significant personal and socioeconomical burden worldwide. Systematic reviews of the prevalence of low back pain (LBP) have estimated that 38.9% of people will experience activity‐limiting pain at some point across their lifetime,^1^ with associated lost economic productivity approximating $296 billion per year in the United States alone.^2^

Spinal pain is often multifactorial and may result from nociception originating from any combination of facet joints, intervertebral discs, muscle, fascia, or vertebral bodies.^3^ Facet joint pain is identified as the primary source of pain in 15% of people with chronic lumbar spine pain and between 54% and 60% of people with cervical pain.^4^ Each facet joint receives dual innervation by the medial branches of the spinal dorsal rami superiorly and inferiorly from adjacent spinal levels.^5^ The medial branch nerves transmit afferent sensory information from spinal segments to higher cortical areas. This includes nociceptive signals from joint trauma, inflammation, or degeneration^6^ and information from stretch receptors found within the joint capsule, which contributes to the body's awareness of joint position, proprioception, and maintenance of balance.^7^, ^8^ Additionally, the medial branch nerve provides efferent motor innervation to spinal stabilizing multifidi. Electromyographic evidence of denervation of the multifidus group has been used to monitor the efficacy of percutaneous radiofrequency ablation (RFA) procedures.^9^

Thus, anesthetic blockade or ablation of the medial branches decreases pain,^10^ reduces afferent sensory and proprioceptive information from the joint and muscles, and also denervates local spinal stabilizing muscles.^4^, ^9^, ^11^ Previous studies investigating the effect of paraspinal denervation on balance in patients with lumbar spinal stenosis have demonstrated that denervation increased patient sway and difficulty controlling center of gravity.^12^ To date, no studies have looked at the relationship between interventional procedures for facetogenic pain and possible implications on balance.

Balance is a dynamic process in which the central nervous system integrates internal and external sensory information from visual, vestibular, and proprioceptive pathways and generates appropriate coordinated motor responses to maintain stability.^13^ Recent advancements in wearable technologies have significantly enhanced quantitative balance assessments. Devices such as the PROTXX Physiological Vibration Acceleration (Phybrata) sensor have demonstrated their utility as a clinical assessment tool to quantify overall balance performance and sensory integration in healthy populations, as well as patient populations with neurophysiologic deficits, such as those with concussion,^14^, ^15^ spinal cord injury,^16^ age‐related balance decline,^17^ or other neurodegenerative conditions such as multiple sclerosis.^18^

In this study, we aimed to determine if there was a difference in postural balance between those pursuing cervical (and specifically C2/3, which has been associated with ataxia when denervated^19^) or lumbar medial branch blocks (MBBs)/RFAs compared to an asymptomatic halthy cohort (HC). We also investigated differences in balance and sensory reweighting prior to and following MBBs/RFAs, and the association with clinical measures of pain intensity (numerical pain rating scale) and balance using the 4‐Stage Balance Test.

## METHODS

### Study design

A quasiexperimental study with matched controls was performed. The study was approved by the University of Calgary Conjoint Health Research Ethics Board (approval REB23‐0330) and conducted in accordance with the Declaration of Helsinki.

### Participants

Participants were recruited from adult patients attending a multidisciplinary chronic pain clinic in Calgary, Alberta, Canada from May 2023 to January 2024. Patients were receiving either diagnostic/prognostic anesthetic MBBs or were undergoing medial branch nerve RFAs of the cervical or lumbar spine.

Further eligibility criteria to determine enrollment suitability included the following: (1) adult individuals aged 18years and older, (2) chronic (>3 months) axial spinal pain involving the cervical or the lumbar spine, and (3) who were receiving either cervical or lumbar MBB/RFA procedures. Participants were ineligible to participate for any of the following exclusion criteria: (1) self‐reported preprocedure orthostatic balance or vestibular impairments; (2) preprocedure symptoms of dizziness, lightheadedness after standing, vertigo, or orthostatic hypotension; (3) cognitive limitations; (4) allergy to local anesthetic or x‐ray contrast dye; or (5) experienced prior MBB/RFA procedural complications (vasovagal episodes, nerve root weakness, etc.).

Age‐/gender‐matched HC (± 1 year) postural data were extracted from the PROTXX database of balance to compare to those with axial spinal pain.^18^

### Experimental procedures

#### Prior to MBB/RFA

Written informed consent was obtained from all participants prior to enrollment. Baseline measures were gathered prior to undergoing standard MBB/RFA procedures. Patients reported their preprocedure level of pain severity on a 0–10 Numeric Pain Rating scale (NPRS). Standard orthostatic vitals were measured in laying and standing positions to ensure no significant influence of postural orthostasis. This was followed by administration of a clinical 4‐Stage Balance Test. The highest level (ie, 1–4) successfully achieved was recorded. Participant balance measures were then recorded using a head‐mounted accelerometer (PROTXX Phybrata sensor) attached to the participant on the right occipital protuberance using two‐sided foam adhesive. Participants were instructed to stand upright in a relaxed position with their arms at their side, maintain a fixed gaze in a straight‐ahead direction, refrain from talking for the period of data collection and maintain as stable balance as possible. They completed a 60‐second period of static balance with feet placed together and with eyes open (EO). After completion of EO standing, a 60‐second static balance assessment was completed with identical instructions except with eyes closed (EC). In both testing scenarios the test administrator stood at the participant's side in case of balance loss. Test data were excluded and trials repeated if there was anomalous participant movement observed (premature EO, coughing/sneezing, etc.) After baseline measures were collected, participants then underwent standard MBB/RFA procedures.

#### Post MBB/RFA

Post procedure, patients repeated the preprocedure protocol. Orthostatic vitals were measured to ensure that no clinically significant orthostatic changes resulted from the procedure.

### Injection Protocol

Diagnostic MBB procedures were performed according to International Pain and Spine Intervention Society guidelines^20^ (Appendix S1). Medial branch RFA was performed using a multitined cannula and perpendicular to the medial branch nerve technique (Appendix S1).

### Outcomes

#### PROTXX Phybrata Accelerometer

The PROTXX Phybrata sensor has been validated as a reliable tool able to measure variance in postural sway.^15^ The Phybrata inertial mass unit includes a three‐axis accelerometer to record *x* (anterior–posterior or front‐back), *y* (vertical), and *z* (medial‐lateral or left–right) acceleration time series data in units of g (ie, head acceleration in each direction due to postural sway). During each 60‐second test, data were recorded at a sampling rate of 100 Hz and filtered to remove drift. For the spectral analysis, the time series signals were calculated using a discrete Fourier Transform to generate a power spectral density (PSD).^14^ The PSD was divided into five specific frequency bands: 0–0.1 Hz (visual regulation, 0.1–0.5 Hz vestibular regulation), 0.5–1 Hz (cortical structures), 1–10 Hz (proprioception and muscle control), and 10–25 Hz (vestibulocollic reflex). Each frequency band was normalized and presented as a percentage contribution to balance control.^14^

The 4‐Stage Balance Test consisted of the participant standing for 10 seconds each in the following positions with EO: feet shoulder width apart, feet together, feet in tandem stance, and standing on one leg. Each 10‐second interval was scored as a pass/fail, and a segment was considered a fail if there was significant postural sway resulting in participant needing to adjust foot position or place their elevated foot on the ground for support. The highest level of successful balance was recorded. This test has shown strong correlation when compared to other scales commonly used to measure for balance in populations being screened for potential fall risk.^20^ Participant failure to maintain balance through tandem stance phase has been associated with increased fall risk.^21^

Pain severity was measured using the numerical rating scale (NRS). Scores ≤5 correspond to mild, scores of 6–7 to moderate, and scores ≥8 to severe pain.^23^ The International Pain and Spine Intervention Society^22^ and the Medicare Local Coverage Determination^24^ use ≥80% reduction in postprocedural reported pain as a standard for the diagnosis of facet‐mediated pain. Many clinicians find this value too restrictive, because it would prevent many patients from potentially beneficial interventional procedures, and therefore have lowered the threshold to a 50% improvement in pain.^25^ Thus, we defined 50% improvement as a categorically significant improvement in clinical symptoms arising from MBB/RFA procedures.

### Sample size

A formal sample size calculation was not performed due to the exploratory nature of the study and the fact that balance in those with axial spinal pain has not been measured using Phybrata data. Thus, a convenience sample that included 30 participants with cervical and 30 participants with lumbar spine pain was planned for initial data collection and to assist with determination of future appropriately powered sample sizes.

### Data analysis

Data were analyzed for normality, and visually and statistically through box plots and Shapiro–Wilk tests. Log transformation was subsequently applied to Phybrata EO and EC data. Descriptive statistics were used to evaluate demographic factors. A series of linear mixed models were constructed to evaluate the fixed main effects of (1) group (MBB/HC), (2) spinal region (cervical/lumbar), (3) C2‐3 (no/yes), and (4) pain category (< 50% vs. ≥50% pain relief post MBBs/RFAs) and the exploratory interactive effects of condition (EO/EC) and time (pre/post MBB/RFA), with random intercepts for participants, on Phybrata sway power. Gender was initially entered in each model and retained if significant. The models were estimated using the restricted maximum likelihood method. Repeated measures analysis of variance calculations were performed to determine changes in sensory weighting across band (0–0.1 Hz, 0.1–0.5 Hz, 0.5–1 Hz, 1–10 Hz, and 10–25 Hz) and pain category (< 50% or ≥ 50%) post MBB/RFA. Normality was assessed using the Shapiro–Wilk test, as well as skewness and kurtosis tests. The repeated measures analysis of variance applies Mauchly's test for sphericity and Geisser–Greenhouse corrections to F, *p* values. Wilcoxon signed ranks test was performed to evaluate the difference in proportions of people fulfilling different levels of the 4‐Stage Balance Test. Spearman's rho tests evaluated the relationship between Phybrata sway power and clinical measures of balance (4‐Stage Balance Test). Pearson's *r* correlation tests were used to evaluate the relationship between Phybrata sway power and (1) age, and (2) pain pre/post MBB/RFA. The strength of the correlation was defined as very small (*r* < 0.1), small (*r* ≤ *r* < 0.3), moderate (0.3 ≤ *r* < 0.5), and large (*r* ≥ 0.5).^26^ Cohen's *d* effect sizes were calculated for the difference in mean sway power between those with axial spinal pain and the asymptomatic HC. The magnitude of the effect size was as follows: small (0.20), medium (0.50), large (0.80), and very large (1.20).^26^ Significance was set at 5%. All analyses were performed using IBM SPSS Statistics for Windows (Version 26.0. Armonk, NY: IBM Corp).

## RESULTS

A total of 61 patients with chronic axial back pain were recruited (33 women and 28 men, mean [± SD] age 58.5 [± 13.6] years). Data for HCs were gender and age matched (± 1 year). Participants had a mean (± SD) duration of spinal pain symptoms of 9.7 (± 10.6) years.

Of the participants recruited, 31 received cervical MBBs/RFAs and 30 received lumbar MBBs/RFAs. One Phybrata dataset did not save correctly and was excluded from statistical analysis. Participants received either a unilateral or bilateral procedure at one, two, or three spinal levels depending on their prior established patterns of pain (Table 1). In the cervical spine, the following levels received interventions: C5/6 (48%), C2/3 (42%), C3/4 (29%), C6/7 (23%), and C4/5 (16%). For the lumbar spine, L4/5 (87%), L5/S1 (87%), L3/4 (20%), and L2/3 (7%) received interventions. There was a significant reduction in mean (± SD) NRS pain pre/post MBB/RFA (NPRS_pre_ = 5.8 (±1.8); NPRS_post_ = 2.6 (±2.4), *p* < .001). Thirty‐seven of the 61 (61%) participants reported >50% pain relief of their axial spinal pain post MBB/RFA.

**TABLE 1:** Axial spinal injection characteristics.

Axial region of spine	Single level (n)	Two levels (n)	Three levels (n)	Unilateral n (%)
Cervical	14	15	2	25 (81%)
Lumbar	4	22	4	12 (40%)

### Correlation between sway power and age and gender

An increase in age was associated with a moderate increase in sway power both pre (*r* = 0.31, *p* = .016) and post MBB/RFA (*r* = 0.45, *p* < .001). Sway power did not differ between genders.

### Differences in sway power between participants with spinal pain and asymptomatic HC

**FIGURE 1:**
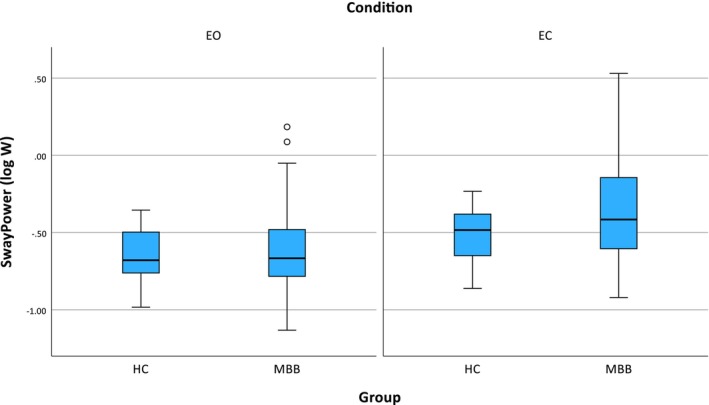
Box plots of log transformation of sway power for participants with spinal pain and healthy controls at baseline (prior to medial branch blocks/radiofrequency ablations). EO/EC, eyes open/eyes closed; HC, healthy cohort; MBB, patient group receiving medial branch blocks/radiofrequency ablations.

Participants with spinal pain demonstrated significantly greater sway power than HC for the EC, but not EO condition (group × condition: *p* < .001; Cohen's *d* EO = 0.36 and EC = 0.73; Figure 1), both prior to and following MBBs/RFAs (group × condition × time: *p* = .83).

**FIGURE 2:**
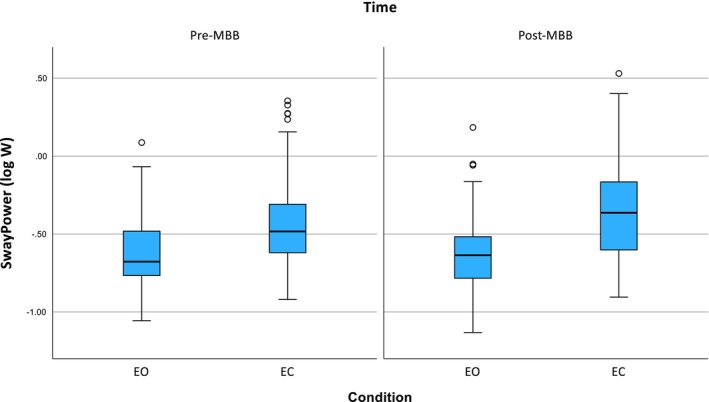
Box plots of log transformation of sway power for participants with spinal pain prior to and following medial branch blocks/radiofrequency ablations. EO/EC, eyes open/eyes closed; MBB, patient group receiving medial branch blocks or radiofrequency ablations.

Differences in balance pre/post MBB/RFA:

1. Phybrata Measures – Following MBBs/RFAs, participants in the EC condition demonstrated a trend toward greater sway power, and those in the EO condition an opposite trend, resulting in an interaction effect (condition × time: *p* = .02; Figure 2). Pairwise comparisons did not demonstrate any significant differences in sway power for either EO (*p* = .28) or EC (*p* = .053) conditions from pre to post MBB/RFA (Cohen's *d* = 0.010 and 0.014 respectively).
2. Clinical Measures – In the 4‐Stage Balance Test, prior to the MBB/RFA 100% (*n* = 61) of participants were able to complete stage 1 (feet apart), 95% (*n* = 58) completed stage 2 (feet offset), 71% (*n* = 43) completed Stage 3 (tandem stance), and 66% (*n* = 40) completed stage 4 (single leg stance). Post MBB/RFA, 100% of participant completed stage 1, 91% completed stage 2, 70% completed Stage 3, and 61% completed stage 4. There was no significant difference in proportions of participants completing each level of the 4‐Stage Balance Test pre and post MBB/RFA (*p* = .23).
3. Association Between Clinical and Phybrata Measures – Prior to MBBs/RFAs, there was a moderate negative relationship between Phybrata sway power and the 4‐Stage Balance Test (*p* = −.38, *p* = .002).

### Differences in sway power between cervical and lumbar axial and C2/3 origin spinal pain

**FIGURE 3:**
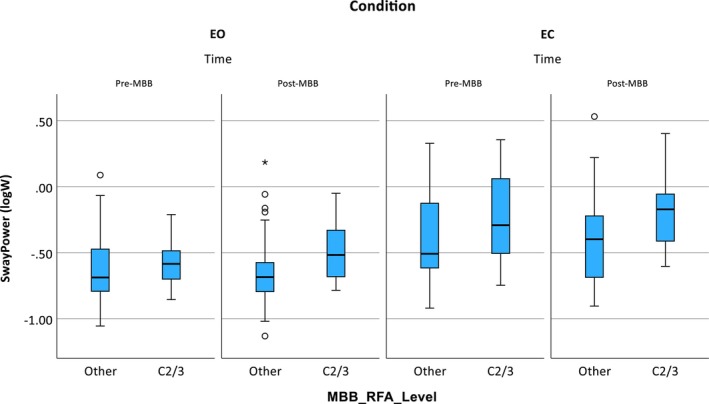
Box plots of log transformation of sway power for participants receiving medial branch blocks/radiofrequency ablations at C2/3 or other spinal levels. EO/EC, eyes open/eyes closed; MBB, patient group receiving medial branch block or radiofrequency ablation; RFA, radiofrequency ablation.

Sway power was not significantly different between those with cervical or lumbar spine pain for EO or EC conditions (spinal region × condition: *p* = .049), either prior to or following MBB/RFAs (spinal region × condition× time: *p* = .87). However, for those receiving C2‐3 interventions, there was a significant increase in sway power following MBB/RFAs (C2‐3 × time: *p* = .028) for both EO and EC conditions (C2‐3 × time × condition: *p* = .88; Figure 3).

### Correlation between sway power and axial spinal pain intensity

There was no significant correlation between Phybrata sway power and NRS for pain, either pre or post MBB/RFA for either EO or EC conditions (Table 2).

**TABLE 2:** Pearson's *r* correlation between Phybrata power sway measures and numerical rating scale of pain intensity prior to and following medial branch blocks/radiofrequency ablations.

		NRS pre MBB/RFA	NRS post MBB/RFA
Protxx EO pre MBB/RFA	Pearson correlation	0.003	−0.241
	Sig. (two‐tailed)	0.980	0.063
	N	60	60
Protxx EC pre MBB/RFA	Pearson correlation	0.043	−0.243
	Sig. (2‐tailed)	0.743	0.061
	N	60	60

Abbreviations: EO/EC, eyes open/eyes closed; MBB, patient group receiving medial branch blocks; NRS, numerical rating scale; RFA, radiofrequency ablation.

### Differences in sway power between responders (≥ 50% relief) and nonresponders (< 50% relief) to MBBs/RFAs

**FIGURE 4:**
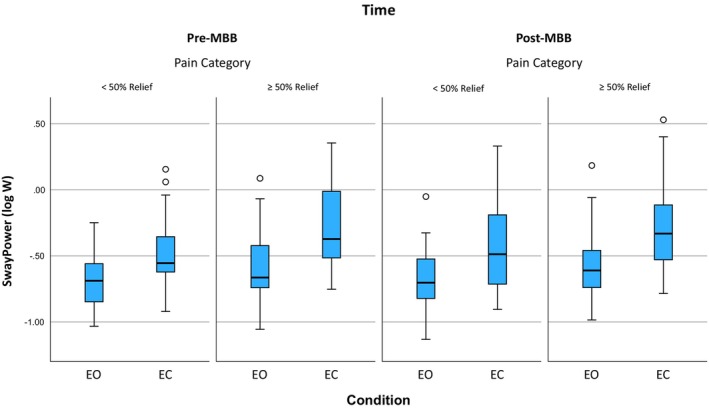
Box plots of log transformation of sway power for participants not responding to (<50% relief) and responding to (≥50% relief) medial branch blocks/radiofrequency ablations. EO/EC, eyes open/eyes closed; MBB, patient group receiving medial branch blocks or radiofrequency ablations.

Responders to MBBs/RFAs demonstrated increased sway power with EC (pain category × condition: Figure 4), both prior to and following MBBs/RFAs (pain category × condition × time: *p* = .46).

### Sensory reweighting: The difference in frequency domain metrics pre/post MBBs/RFAs for both nonresponders and responders

**FIGURE 5:**
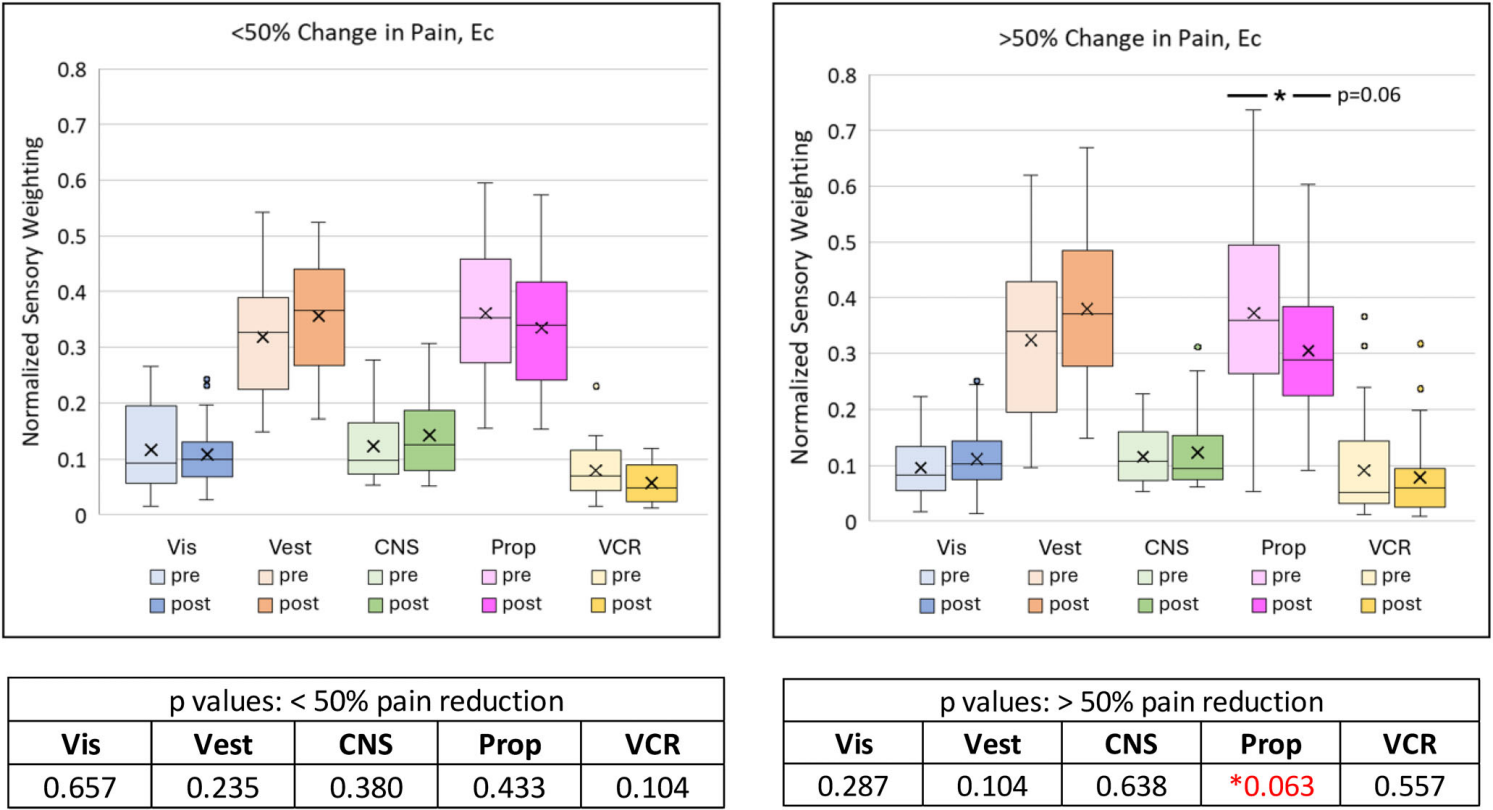
Power spectral density box plots pre/post medial branch blocks/radiofrequency ablations for different frequency bands for those who demonstrated (A) < 50% pain reduction and (B) ≥ 50% pain reduction. CNS, central nervous system; Prop, proprioceptive; Vis, visual; Vest, vestibular; VCR, vestibulocollic reflex.

There was no significant effect of frequency band for nonresponders to MBBs/RFAs (<50% relief of pain post MBB/RFA; Figure 5A). There was a nonsignificant trend toward sensory reweighting for those reporting significant pain relief, with a reduction in the proprioceptive contribution to balance control post MBB/RFA (*p* = .06; Figure 5B).

## DISCUSSION

To our knowledge, this study is the first to investigate the effect of medial branch interventional procedures on balance, measured using Phybrata sensors, in patients with chronic axial spinal pain. For the primary aim, participants with axial spinal pain demonstrated reduced balance (greater sway power) compared with HC for the EC condition. Further analysis revealed that those who experienced ≥50% pain relief following MBBs/RFAs (ie, facetogenic source of pain) demonstrated increased postural sway with EC both before and after the procedure. Patients receiving C2/3 interventions demonstrated increased postural sway following the procedure, irrespective of eyes being open or closed, which is consistent with studies reporting patient development of ataxia post‐C2/3 RFA.^19^ The remainder of patients did not demonstrate changes in balance following MBBs/RFAs, regardless of pain relief, suggesting that the anesthetization of spinal muscular proprioceptors at C2/3 has a greater association with postural control. Overall, participants who initially exhibited balance deficits continued to show reduced postural stability even after MBB/RFA procedures. Interestingly, those with presumed facet joint pain (≥ 50% relief) demonstrated a non‐significant trend (*p* = .06) toward sensory reweighting, with PSD analysis demonstrating less reliance on the proprioceptive system. In addition, this study demonstrated that Phybrata measures were associated with clinical measures of balance. Not surprisingly, Phybrata sway power was positively associated with age. Phybrata measures did not differ between genders, regions of spinal pain (cervical vs. lumbar), and were not associated with pain intensity.

Our study investigated balance using a head‐mounted Phybrata sensor, which has previously been validated as an effective tool for measuring balance.^18^, ^27^ It is well established that individuals with chronic neck and LBP often experience impaired balance.^28^, ^29^ The mechanisms underlying these balance deficits are incompletely understood. Balance is a complex, whole‐body response that requires coordinated integration between afferent input and efferent output systems. Thus, dysfunction of any of these systems could influence balance. The Phybrata sensor offers the advantage of measuring different frequency domains corresponding to different postural control subsystems (eg, visual, vestibular, proprioceptive sensory inputs, and central nervous system integration) associated with maintaining balance.^18^, ^19^, ^27^ The study investigated axial spinal pain, with participants equally divided between the cervical and lumbar spine regions and both genders. All participants presented with a chronic history of moderate‐to‐severe pain. Notably, ∼60% of participants reported >50% pain relief following the MBBs/RFAs, suggesting that the majority presented with facet joint nociception, consistent with studies investigating the prevalence of spinal facet‐mediated pain.^30^, ^31^, ^32^, ^33^ It is well documented that facet‐mediated LBP increases with age,^32^ whereby at age 60, it becomes the predominant anatomical source of axial lumbar spine pain.^31^, ^32^ Thus, the studied sample is representative of other studies evaluating the prevalence of axial spinal pain and these results should be generalizable to those with persistent axial spinal pain undergoing interventional procedures. Additionally, the Phybrata data were associated with the 4‐Stage Balance Test and correlated with age, supporting the utility and generalizability of results.

In people with axial spinal pain, both peripheral sensitization and central pain mechanisms can result in aberrant proprioceptive influence and subsequent balance deficits.^34^ Pain and local tissue damage can directly influence proprioception as evidenced in athletes with chronic ankle sprains.^35^ Pain may also result in aberrant proprioceptive input due to a hesitation to move to avoid further exacerbation of pain.^36^, ^37^ It has also been postulated that central processing of proprioceptive information is obstructed by competing nociceptive pathway signals, thus impairing central awareness of posture change.^38^ Prior studies describing these associations failed to clarify the underlying source of LBP of participants. A recent meta‐analysis reported that of 43 studies looking at kinematic changes in participants with LBP, 85% of studies failed to provide diagnostic criteria for LBP or described LBP as “nonspecific.”^39^ As such, although it is established that LBP can lead to proprioceptive changes of the trunk, there is a current gap in the literature regarding the respective contributions of various sources of nociception to that deficit (eg, facetogenic vs. discogenic). Our data at least suggest that those with facet‐mediated pain (≥50% response to MBBs/RFAs) demonstrate reduced balance.

When evaluating our primary analysis of sway power, our data did not support a relationship between pain intensity level and balance. Of important note, our sample of participants were those who, after assessment of their presenting symptom of LBP, had clinical features most suggestive of facetogenic pain. Our study therefore did not include patients with presentations in keeping with discogenic, radicular, or sacroiliac joint dysfunction. Our study found pain intensity was not associated with balance, either prior to or following MBBs/RFAs. Similarly, improvements in pain were not correlated with improvements in balance. This may not be surprising, as short‐term pain reduction, as measured in this study, does not necessarily result in an immediate change of movement pattern and hence change in proprioceptive input. Patients with longstanding pain will have likely developed learned movement patterns that minimize exacerbation of pain symptoms. These learned movement patterns may be at the expense of proprioceptive input, as this study showed through the difference in balance between those with axial spinal pain and HCs. Therefore, changes in balance measures post MBB/RFA (and with associated reduced proprioceptive input and pain reduction) may be minimal as the body has already adapted to a reduced reliance on proprioceptive input–excepting when procedures were performed at C2/3, which results in partial denervation of the semispinalis capitis muscle and is important for head/neck postural control, given its interaction with the suboccipital muscles and their high concentration of proprioceptors.^40^ Overall, the body is able to adopt strategies to maintain balance based on expectation, goals for the movement task, prior experience, and environmental context. Hence, the body is likely able to adapt by compensating for loss or change in proprioceptive balance input information^41^ as observed in this study post‐MBB/RFA with the trend toward sensory reweighting involving the vestibular system.

Our study was not powered to detect differences in each of the frequency bands associated with balance. Of particular note was the reduction in proprioceptive control (frequency band 1–10 Hz) associated with balance in those who reported a successful response to MBBs/RFAs (≥ 50% pain reduction). Although not reaching significance, sensory reweighting was observed, with a demonstrated reduction in the proprioceptive contribution to balance control, and a corresponding trend toward increased vestibular contributions. Thus, although overall balance performance was not significantly changed in those with facet‐mediated pain (≥ 50% pain reduction), anesthetization of the medial branches and associated muscular proprioceptors in the neck resulted in a change in sensory weighting used to maintain that balance. Despite the proprioceptors being similarly anesthetized in participants whose pain was nonfacetogenic (< 50% response), there was no evidence of sensory reweighting. Thus, facet‐mediated pain was shown to influence balance through reintegration of balance systems, independent of surrounding proprioceptive input.

Clinically, these results are reassuring from a patient safety perspective, suggesting that the risk of introducing new balance impairments to patients not receiving C2/3 MBB/RFAs is low. This helps in patient counseling around procedure risks, because the results indicate that from a balance perspective, they should be able to engage in regular daily activities without being at increased risk of postural instability or falls. On the other hand, for those individuals with degraded balance compared to HCs, MBBs/RFAs did not improve their balance, whether they received significant pain relief (≥ 50%) or not. Thus, these results draw attention to the need to restore balance in patients with chronic axial spinal pain of facetogenic origin in the cervical or lumbar spine regions.

There are several factors that require further discussion. This study investigated balance in static standing for 1 minute each of EO and EC conditions. We are unable to comment on the influence of MBBs/RFAs and pain reduction on changes in more dynamic balance evaluations and gait mechanics or in more fatiguing situations. It is also possible that the response to single MBBs performed were false positives, as demonstrated in prior studies,^31^, ^42^ thus diluting the influence of pain reduction on balance measures. Repeating the measures following a second MBB would provide further clarification on the role of facet‐mediated pain and balance measures. We also did not screen for mild visual, vestibular, or peripheral proprioceptive (eg, history of ankle sprains, diabetic neuropathy) deficits. Although speculative, people with such deficits may rely more heavily on their spinal proprioceptive control of balance, and as such, anesthetizing spinal proprioceptors may result in greater balance changes post MBB/RFA with less ability to compensate. This topic requires further research. Due to low participant numbers for subcategories, we could not confidently investigate the influence of multiple level anesthetic blockade or unilateral versus bilateral procedures, which could have influenced outcomes. The volume of anesthetic varies with multiple levels, and safety considerations limit denervation of the medial branches when performing RFA to less than three levels to prevent complications arising from theoretical spinal destabilization. Thus, further research with larger samples is warranted.

In conclusion, this study demonstrated that balance, as measured with a head‐mounted Phybrata sensor, is impaired in those with axial spinal pain of facetogenic origin when compared to asymptomatic HCs, particularly in those with primary facetogenic pain (ie, respond to MBBs/RFAs). Anesthetizing the medial branches resulted in increased postural sway when the procedures were performed at C2/3. Otherwise, this did not translate to significant changes in balance deficit, nor did the reduction of pain lead to improvement in balance, illustrating that patients rapidly adapted to this loss of proprioception through sensory reweighting, providing reassurance to patients that their overall balance performance will not worsen if undergoing MBBs/RFAs for axial spine pain.

## DISCLOSURE

John Ralston is the founder and CEO of PROTXX, Inc. and has a financial interest in the company. The remainder of the authors have no conflicts of interest to disclose.

## ETHICS STATEMENT

Data are available upon request. Ethical clearance for this study was granted by the University of Calgary Research Ethics Committee (REB21‐0919) and performed according to the Declaration of Helsinki.

This journal‐based CME activity is designated for 1.0 *AMA PRA Category 1 Credit* ^TM^. Effective January 2024, learners are no longer required to correctly answer a multiple‐choice question to receive CME credit. Completion of an evaluation is required, which can be accessed using this link, https://onlinelearning.aapmr.org/. This activity is FREE to AAPM&R members and available to nonmembers for a nominal fee. CME is available for 3 years after publication date. For assistance with claiming CME for this activity, please contact (847) 737–6000. All financial disclosures and CME information related to this article can be found on the Online Learning Portal (https://onlinelearning.aapmr.org/) prior to accessing the activity.

## Supporting information


**Appendix S1.** Supporting Information.
