# Supplementary material for: Analysis of balance in adults with chronic axial spinal pain receiving medial branch block or radiofrequency ablation procedures
Source: PM R. 2026 Jan 19;18(8):863–73. doi: 10.1002/pmrj.70083 (PMC13476662; doi:10.1002/pmrj.70083)
Supplement: Supplementary file 1 [file PMRJ-18-863-s001.docx]

**Appendix:**

Fluoroscopically guided MBBs of the cervical facet joints were performed most often with the patients in a side lying position. With superimposed right and left lateral masses, the medial branch nerves of C3 through C6 were blocked at the midpoint of the articular pillar. C7 was blocked at the superior articular process-transverse process junction. The dual innervations of each facet joint was targeted with blocks (eg. For the C5/6 facet joint, both the 5th and the 6th cervical medial branches were blocked). Before local anesthetic injection, a small amount of x-ray contrast media was injected to ensure that the needle tip was not intravascular and that the injectate flow would cover the anticipated location of the target medial branch nerve. 0.3mL of local anesthetic was injected per C3-7 MBB. If the lower cervical lateral masses were not clearly visible with the patient in a side lying position, the patient was positioned prone and the midpoint of the lateral mass was approached using posterior to anterior trajectory. For C2/3, 0.3 mL of local anesthetic was injected at the joint line followed by 0.3mL of local anesthetic just above and just below the joint line to anesthetize the 3^rd^ occipital nerve.

Fluoroscopically guided MBBs of the lumbar facet joints were performed with the patient in a prone position. The C-arm was positioned in 15^o^-25^o^ of oblique angulation ipsilateral to the target site in order to bring the superior articular process and transverse process junction into view (“Scottie-dog” view). The L1 through L4 medial branch nerves were blocked at the midpoint of the root of the superior articular process and the dorsal surface of the transverse process. For each facet joint, the dual contributing medial branch nerves were blocked (eg. For the L4/5 facet joint, both the 3rd and 4th lumbar medial branch nerves were blocked). The L5 posterior primary ramus, was targeted as it courses over the intersection of the S1 superior articular process and the superior edge of the sacral ala.  In similar fashion to the cervical blocks, a small amount of x-ray contrast media was injected to ensure optimal nonvascular injectate flow. 0.5mL of local anesthetic was injected at each lumbar target site.
